# P-1461. Influenza Vaccine Amongst Persons Experiencing Homelessness: Evaluating Attitudes, Barriers, and Trust

**DOI:** 10.1093/ofid/ofae631.1633

**Published:** 2025-01-29

**Authors:** Yasmeen Mann, Angela Ishak, Ugonna K Ogbenna, Lauren Kasmikha, Medha R Cherabuddi, Katherine Joyce, Charlalynn Harris, Mariia Numi, Maria Santana-Garcés, Najibah K Rehman, Richard Bryce, Seema Joshi, Marcus Zervos

**Affiliations:** Henry Ford Hospital, Detroit, Michigan; Henry Ford Hospital, Detroit, Michigan; Cook County Health System, Southfield, Michigan; Wayne State University School of Medicine, Detroit, Michigan; Henry Ford Hospital, Detroit, Michigan; Henry Ford Hospital, Detroit, Michigan; Henry Ford Health System, Duluth, Georgia; Henry Ford Hospital, Detroit, Michigan; Henry Ford Health, Livonia, Michigan; Henry Ford Health System, Duluth, Georgia; Henry Ford Hospital, Detroit, Michigan; Henry Ford Hospital, Detroit, Michigan; Henry Ford Hospital, Detroit, Michigan

## Abstract

**Background:**

In Detroit, persons experiencing homelessness (PEH) have been a vulnerable part of society due to their comorbidities and unique barriers in access to care. Despite PEH experiencing higher rates of vaccine-preventable diseases, there is a lack of information on strategies to improve vaccine rates. The objective of this project is to obtain an understanding of enablers and barriers in influenza vaccine uptake amongst PEH, to inform future work to increase vaccine uptake.

**Figure:**
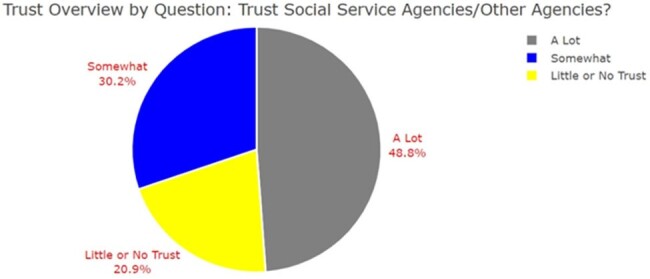

**Methods:**

This is a cross-sectional study of survey data from adult PEH encountered during Street Medicine (SM) outreach in Detroit to assess enablers and barriers in influenza vaccination. Respondents were provided a complimentary bus pass for participation. Descriptive statistics were conducted on sociodemographic variables with analysis of variables related to level of homelessness, vaccine concerns, vaccination status, and underlying health problems.

**Figure:**
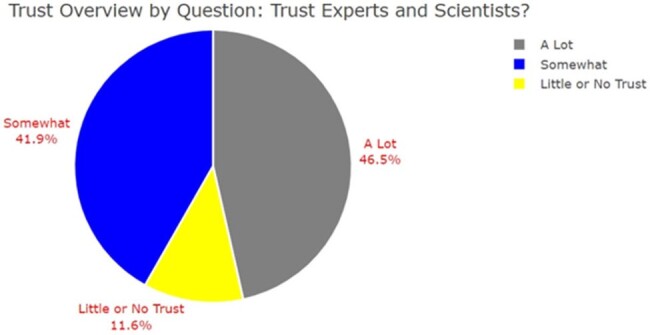

**Results:**

43 participants' surveys were analyzed. Participant vaccine status and level of homelessness varied with majority (35%) reporting spending at least 1 night in a tent or other dwelling. Regarding participant demographics, 86% (n=37) were male and 81% (n=35) were Black or African American (AA) (Table 1). Amongst survey respondents, the highest sources of trust for information regarding influenza vaccine was found amongst: ‘Social Services Agencies or Other Services’ 95.2% (n=20) and ‘Experts and Scientists’ 93.8% (n=15) (Figures 1 and 2). Variables regarding sources of information trusted were analyzed via chi-square analysis to evaluate for statistical significance (p-value < 0.05) amongst demographic variables (Figure 3).

**Figure:**
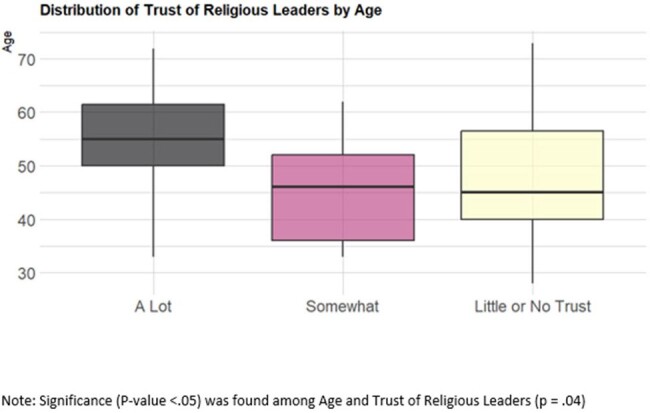

**Conclusion:**

Previous literature regarding attitudes towards vaccines amongst PEH has demonstrated distrust in healthcare. Results of this study are unique in identifying healthcare members as resources trusted by most participants in providing information on vaccines. This emphasizes the importance of SM outreach in Detroit in building trusting relationships with PEH in addition to decreasing barriers in access to care, to help target future vaccination initiatives.

**Table 1:**
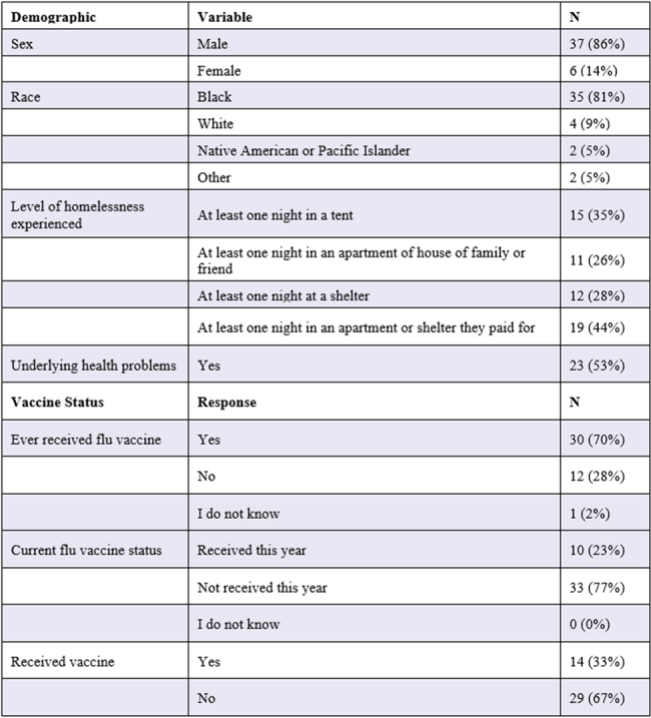
Participant Demographics and Influenza Vaccination Status

**Disclosures:**

**Seema Joshi, MD**, AbbVie: Grant/Research Support **Marcus Zervos, MD**, Johnson and johnson: Grant/Research Support|Moderna: Grant/Research Support

